# Insomnia with physiological hyperarousal is associated with lower weight: a novel finding and its clinical implications

**DOI:** 10.1038/s41398-021-01672-5

**Published:** 2021-11-28

**Authors:** Rong Ren, Ye Zhang, Linghui Yang, Larry D. Sanford, Xiangdong Tang

**Affiliations:** 1 Sleep Medicine Center, Department of Respiratory and Critical Care Medicine, Translational Neuroscience Center, State Key Laboratory, West China Hospital, Sichuan University, Chengdu, China; 2 Sleep Research Laboratory, Center for Integrative Neuroscience and Inflammatory Diseases, Department of Pathology and Anatomy, Eastern Virginia Medical School, Norfolk, VA USA

**Keywords:** Psychiatric disorders, Physiology

## Abstract

Previous studies on the association of insomnia with body mass index (BMI) have been controversial. Physiological hyperarousal, the key pathological mechanism of insomnia, may be an important reason for different findings. We explored whether insomnia with physiological hyperarousal measured by the multiple sleep latency test (MSLT) is associated with body-weight differences. A total of 185 normal sleepers and 440 insomniacs were included in this study. Insomnia was defined by standard diagnostic criteria with symptoms lasting ≥6 months. All subjects underwent one night of laboratory polysomnography followed by a standard MSLT. We used the median MSLT value (i.e., ≥14 min) to define physiological hyperarousal. BMI was based on measured height (cm) and weight (kg) during the subjects’ sleep laboratory visit. BMI > 25 kg/m^2^ was defined as overweight, while BMI < 18.5 kg/m^2^ was defined as underweight. After controlling for confounders, the odds of lower weight rather than overweight were significantly increased among insomnia patients with increased MSLT: insomnia with MSLT 14–17 min and MSLT > 17 min increased the odds of lower weight by approximately 89% (OR = 1.89, 95% CI 1.00–4.85) and 273% (OR = 3.73, 95% CI 1.51–9.22) compared with normal sleepers, respectively. In contrast, insomnia in patients with MSLT 11–14 min and 8–11 min was not different from normal sleepers in terms of body weight. Insomnia associated with physiological hyperarousal, the most severe phenotype of chronic insomnia, is associated with higher odds of lower weight and underweight compared with normal sleepers. This is a novel finding consistent with previous physiologic data and has significant clinical implications.

## Introduction

Insomnia is one of the most prevalent sleep disorders and it has been recognized as a major public health issue. About 30–35% of the population have experienced symptoms of insomnia, and at least 10% meet the clinical diagnostic criteria of chronic insomnia [1]. Insomnia is associated with public health implications and medical comorbidity, including impaired occupational performance, worse quality of life, hypertension, and diabetes [2]. Instead of a disorder of impaired nocturnal sleep, insomnia is now characterized as a disorder of 24-h physiological hyperarousal. This hyperarousal has been shown in a number of physiological measures, including activation of the HPA axis, increased secreted catecholamines [3, 4], increased basal metabolic rate [5], increased body temperature [6], altered heart rate [7], and cortical activation observed in functional neuroimaging [8].

Physiological hyperarousal is reported to be present in 50% of chronic insomniacs and associated with higher risk of neurocognitive impairment, hypertension, diabetes mellitus, and mortality when compared with chronic insomniacs without physiological hyperarousal [9]. Thus, physiological hyperarousal may be the key factor leading to increased risk for medical disorders and mortality in this insomnia phenotype [10]. Accordingly, Vgontzas and colleagues [9] have proposed two subtypes of insomnia. One is characterized by physiological hyperarousal, resulting in substantial medical and neurocognitive morbidity, and the other is defined by a more normal sleep pattern and lower morbidity rates. Among all measures of physiological hyperarousal, objective sleep duration from PSG and mean sleep latency from the multiple-sleep-latency test (MSLT) are the most available and useful tools in routine clinical practice. MSLT is a lab-based tool designed to evaluate objective daytime sleepiness, physiologic sleep propensity, and arousal [11, 12]. Lower MSLT values suggest excessive daytime sleepiness (e.g., MSLT < 8 min) [13], whereas higher values indicate physiological hyperarousal [12].

Abnormal body weight, especially overweight and obesity, are increasing problems in industrial societies [14]. Numerous studies have found a significant relationship between short sleep duration and obesity [15, 16]. A systematic review has also found that poor sleep quality itself is related to greater risk of obesity [17]. It is thus logical to expect insomnia to be associated with greater risks of overweight and obesity, since subjective sleep impairment and short sleep duration are commonly seen in some insomniacs. However, previous studies exploring the association of insomnia and body mass index (BMI) have reported inconsistent results [1, 18]. In a prospective study with 812 subjects who were free of metabolic syndrome at baseline, insomnia did not predict the development of metabolic syndrome, including overweight three years later [19]. Cronlein et al. [20] even found a lower BMI in 233 severe-insomnia patients when compared with data from the general population. On the other hand, Mysliwiec et al. [21] found a slightly higher BMI in insomnia when exploring rates of comorbid sleep disorders in 110 military personnel. A recent meta-analysis conducted by Chan [1] did find a small but significant cross-sectional correlation between insomnia symptoms and BMI, although the odds of having obesity were not significantly greater in patients with an insomnia diagnosis. One explanation for these inconsistent findings may be that no study has examined the potential modifying effect on weight of the insomnia-specific hyperarousal phenotype, which is associated with increased basal metabolic rate and possible consequent lowering effect on weight if intake of calories stays constant [22]. Thus, in this study, we examined the association between physiological hyperarousal, as measured by the MSLT and BMI in insomnia patients. We hypothesized that physiological hyperarousal is an effect modifier in the relationship between insomnia and BMI.

## Subjects and methods

### Subjects

This was a cross-sectional, observational study conducted at the Sleep Medicine Center, West China Hospital. The study procedure was approved by the University’s Institutional Review Board and informed consent was obtained from each participant.

All patients with insomnia were Chinese adults (aged >18 years) and were selected consecutively from the Sleep Medicine Center, West China Hospital, between Jan 2010 and Dec 2019. Insomnia was diagnosed by five psychiatrists in our center during clinical interviews based on Diagnostic and Statistical Manual for Mental Disorders (DSM-IV-TR) criteria and symptoms lasting ≥6 months [23]. College students, the medical and technical staff, and visitors of West China Hospital were recruited to serve as normal sleeper controls by posters during the same period. Normal sleepers were adults who had no sleep problems, major medical, or psychiatric conditions based on their history and physical examination. Individuals with insomnia and normal sleep were matched for age and sex within the study sample with an initial ratio of 4:1 (insomnia patients to controls). We then excluded subjects who had (1) a chronic sleep-disruptive medical condition (e.g., pain); (2) a current major physical or psychiatric condition (e.g., arrhythmia and depression); (3) current or recent (within the past three months) use of hypnotics, antidepressants, anxiolytics, or antipsychotics; (4) evidence of a sleep-disordered breathing disorder (apnea–hypopnea index ≥5 events/h) and/or sleep-related movement disorder (periodic limb-movement index ≥15 events/h); (5) evidence of a hypersomnia disorder for normal sleepers, that is, MSLT ≤ 8 min, ≥2 sleep-onset rapid eye-movement periods; we also exclude all insomnia patients who have a MSLT ≤ 8 min; and (6) other comorbid sleep disorders (e.g., restless-legs syndrome).

During the recruitment period, 2183 subjects were studied in the sleep laboratory. After an overnight polysomnography and MSLT, a total of 625 individuals, 440 insomnia patients, and 185 normal sleepers met the selection criteria for the present study.

### Polysomnography

All subjects were evaluated for a full-night PSG in the sleep laboratory (Alice 5 Diagnostic Sleep System; Philips Respironics, Bend, OR, USA). During this evaluation, subjects were allowed to sleep ad libitum based on their routine sleep time. All sleep parameters recorded were analyzed and scored according to the American Academy of Sleep Medicine criteria by senior PSG technicians [24].

### Multiple sleep latency test

The MSLT was performed on the day immediately after the overnight PSG and comprised four 20-min nap opportunities at intervals of 2 h (9:00, 11:00, 13:00, and 15:00). Sleep onset required the presence of any sleep stage for the duration of ≥30 s. If no sleep occurred, the trial was terminated at 20 min and a sleep latency of 20 min was assigned. The mean sleep latency was the average sleep latency from all four naps. Then, we divided insomnia patients into quartiles based on MSLT (>17 min, 14–17 min, 11–14 min, and 8–11 min). We defined subjects with MSLT > 14 min as having physiological hyperarousal.

### Anthropometric measurements

All anthropometric measures were obtained during the sleep laboratory visit. Body weight and height were measured after urination by a medical height and weight scale (Shengyuan HGM 600, China) with light clothing and no shoes. BMI was calculated as body weight (in kg) divided by the square of height (in meters). We modeled BMI as a 3-level variable: underweight (BMI < 18.5 kg/m^2^), normal weight (BMI 18.5–25 kg/m^2^), and overweight (BMI > 25 kg/m^2^) [25]. Neck circumference (NC) and waist circumference (WC) were measured with an inelastic, flexible tape to the nearest 1 mm. NC was measured from the level just below the laryngeal prominence perpendicular to the long axis of the neck with head positioned upright, while WC was measured as the circumference midway between the lower ribs and the iliac crest in a standing position. NC and WC were expressed in cm.

### Other key measurements

Subjective daytime sleepiness was measured by the Epworth Sleepiness Scale [26] that subjects completed during the sleep laboratory visit. Diabetes mellitus was defined as whether subjects had a physician diagnosis per clinical history or were being treated for diabetes mellitus. We also ascertained the history of use of tobacco (current or past use of any type of tobacco product), alcohol (>2 alcohol drinks per day), and caffeine (>2 coffee or tea drinks per day).

### Statistical analyses

Data are presented as the mean ± SD for continuous variables and frequency and percent for categorical variables. Differences in sample characteristics according to insomnia status and different levels of hyperarousal were assessed using independent-sample t-tests, ANOVA, or Mann–Whitney U tests for normally distributed and not normally distributed continuous variables, respectively. Chi-square tests were used for categorical variables.

Logistic regression was used to assess the independent association between insomnia and physiological hyperarousal (based on MSLT) with underweight and overweight. The covariates we adjusted for included major confounding factors expected to affect this relation, such as age, sex, and total sleep time. First, we separately studied the association of underweight and overweight with insomnia. Second, we divided samples into 5 groups based on insomnia and the MSLT values: normal sleepers, insomniacs with an MSLT > 17 min, insomniacs with an MSLT 14–17 min, insomniacs with an MSLT 11–14 min, and insomniacs with an MSLT 8–11 min. Then, we evaluated logistic-regression models that included 5 groups using normal sleepers as a reference group. We calculated the adjusted odds ratios (ORs) and the 95% confidence intervals (95% CIs) while adjusting for age, sex, tobacco, alcohol drinking, and coffee use, diabetes mellitus, AHI, total sleep time, sleep efficiency, and arousal index to estimate the odds of underweight and overweight associated with insomnia and physiological hyperarousal.

In order to further characterize the association between different levels of physiological hyperarousal groups and BMI, NC, and WC, we conducted an ANCOVA. Post hoc Dunnett tests were used to control for type-I errors in multiple comparisons. Linear-regression models were used to explore the association between MSLT values and BMI, NC, and WC in normal sleepers and insomniacs, respectively. The covariates we adjusted for included age, sex, tobacco, alcohol drinking, and coffee use, diabetes mellitus, AHI, total sleep time, sleep efficiency, and arousal index.

Data were analyzed using SPSS 19.0. Comparisons with *p*-values < 0.05 were considered statistically significant.

## Results

A total of 70 individuals were underweight and 129 individuals were overweight. The overweight group was the oldest (42.00 ± 11.77), had the shortest sleep-onset latency (21.24 ± 38.62), and the shortest MSLT values (11.12 ± 4.74). The underweight group was the youngest (33.55 ± 13.25), had the longest sleep-onset latency (28.78 ± 25.97), and the longest MSLT values (15.29 ± 3.41). The group with normal weight had medium age (41.59 ± 11.01), sleep-onset latency (26.34 ± 40.56), and MSLT values (13.05 ± 4.65). No differences in total sleep time, sleep efficiency, arousal index, sleep architectures, AHI, and ESS were obtained between the three groups.

The demographic, clinical, and sleep characteristics of normal sleepers and insomnia are presented in Table 1. Table 2 presents the demographic, clinical, and sleep characteristics of insomnia subgroups based on different levels of physiological hyperarousal. We also showed BMI, neck circumference, and waist circumference of normal sleepers with different levels of MSLT in Table S1.

**Table 1:** Demographic, clinical and sleep characteristics of study sample.

Characteristics	Normal sleep (*n* = 185)	Insomnia (*n* = 440)	*P*
Demographic and clinical			
Men, *n* (%)	71 (38.4)	156 (35.5)	0.470
Age (years)	41.14 ± 11.45	39.22 ± 10.64	0.088
Underweight, *n* (%)	8 (4.3)	62 (14.1)	0.003
Overweight, *n* (%)	61 (33.0)	68 (15.5)	<0.001
Body mass index (kg/m^2^)	23.72 ± 3.45	21.80 ± 2.72	<0.001
Neck circumference (cm)	34.82 ± 4.06	33.32 ± 3.38	<0.001
Waist circumference (cm)	83.98 ± 10.18	78.76 ± 9.04	<0.001
Diabetes mellitus (%)	3 (1.6)	4 (0.9)	0.680
Smoking, *n* (%)	25 (13.5)	51 (11.6)	0.790
Alcohol drinking, *n* (%)	36 (19.5)	51 (11.6)	0.033
Coffee using, *n* (%)	28 (15.1)	34 (7.7)	0.020
Nighttime sleep			
Sleep onset latency (min)	16.39 ± 18.69	26.44 ± 33.67	0.001
Total sleep time (min)	418.27 ± 67.61	397.44 ± 81.57	0.001
Sleep efficiency (%)	83.52 ± 10.82	78.19 ± 14.86	<0.001
Arousal index (events/h)	18.53 ± 10.43	18.87 ± 9.95	0.620
N1 (% TST)	16.48 ± 8.89	17.45 ± 12.21	0.301
N2 (% TST)	52.08 ± 10.06	52.08 ± 12.28	0.970
N3 (% TST)	12.12 ± 7.61	12.88 ± 8.02	0.230
REM (% TST)	19.31 ± 5.46	17.58 ± 6.29	0.001
AHI (events/h)	2.11 ± 1.49	1.23 ± 1.23	<0.001
Daytime alertness			
ESS	6.89 ± 5.44	4.33 ± 4.73	<0.001
MSLT (min)	11.38 ± 4.66	14.19 ± 3.48	<0.001
9:00 nap (min)	11.11 ± 6.73	13.75 ± 6.43	<0.001
11:00 nap (min)	10.58 ± 6.79	13.82 ± 6.20	<0.001
13:00 nap (min)	11.63 ± 6.86	13.34 ± 6.27	<0.001
15:00 nap (min)	11.62 ± 6.84	15.22 ± 6.00	<0.001

Percent for categorical and other variables are presented with mean ± SD.

**Table 2:** Demographic, clinical and sleep characteristics of insomnia with different level of hyperarousal.

Characteristics	8 min < MSLT < 11 min (*n* = 99)	11 min ≤ MSLT < 14 min (*n* = 118)	14 min ≤ MSLT ≤ 17 min (*n* = 115)	MSLT > 17 min (*n* = 108)	*P*
Demographic and clinical					
Men, *n* (%)	45 (45.5%)	36 (30.5%)	42 (36.5%)	33 (30.6%)	0.084
Age (years)	38.36 ± 10.27	39.03 ± 9.80	39.25 ± 10.38	40.18 ± 12.11	0.670
Underweight, *n* (%)	7 (7.1%)	14 (11.9%)	14 (12.2%)	27 (25.0%)	0.002
Overweight, *n* (%)	19 (19.2%)	18 (15.3%)	18 (15.7%)	13 (12.0%)	0.004
Body mass index (kg/m^2^)	22.32 ± 2.71	21.90 ± 2.72	21.54 ± 2.55	21.19 ± 2.81	0.030
Neck circumference (cm)	33.38 ± 3.42	33.16 ± 3.06	33.60 ± 3.50	33.14 ± 3.59	0.790
Waist circumference (cm)	79.81 ± 11.00	79.79 ± 7.68	77.79 ± 9.03	77.53 ± 8.03	0.180
Diabetes mellitus (%)	1 (1.1%)	1 (0.9%)	1 (0.9%)	1 (1.0%)	0.990
Smoking, *n* (%)	18 (18.9%)	9 (8.3%)	11 (10.4%)	13 (13.1%)	0.130
Alcohol drinking, *n* (%)	15 (15.8%)	14 (12.8%)	12 (11.3%)	10 (10.1%)	0.670
Coffee using, *n* (%)	7 (7.4%)	11 (10.1%)	7 (6.7%)	9 (9.0%)	0.800
Nighttime sleep					
Sleep onset latency (min)	22.08 ± 27.52	23.03 ± 27.51	27.76 ± 36.98	32.79 ± 39.99	0.075
Total sleep time (min)	416.18 ± 61.67	402.61 ± 85.27	395.66 ± 86.03	376.51 ± 84.73	0.005
Sleep efficiency (%)	83.20 ± 9.79	79.55 ± 15.28	77.27 ± 15.48	73.08 ± 15.98	<0.001
Arousal index (events/h)	21.26 ± 9.24	19.20 ± 10.50	18.13 ± 10.21	17.09 ± 9.32	0.019
N1 (% TST)	17.92 ± 12.36	17.37 ± 11.56	18.37 ± 14.94	16.12 ± 9.22	0.550
N2 (% TST)	51.62 ± 12.20	51.94 ± 12.38	51.26 ± 13.48	53.54 ± 10.88	0.530
N3 (% TST)	12.18 ± 7.36	12.98 ± 7.88	13.00 ± 8.79	13.30 ± 7.96	0.780
REM (% TST)	18.29 ± 5.68	17.69 ± 6.83	17.38 ± 6.63	17.04 ± 5.86	0.530
AHI (events/h)	1.17 ± 1.17	1.27 ± 1.08	1.29 ± 1.46	1.17 ± 1.18	0.820
Daytime alertness					
ESS	5.27 ± 5.44	4.46 ± 4.75	4.12 ± 4.39	3.55 ± 4.25	0.080
MSLT (min)	9.59 ± 0.90	12.40 ± 0.87	15.57 ± 0.88	18.73 ± 1.13	<0.001
9:00 nap (min)	9.04 ± 5.65	10.69 ± 6.04	16.05 ± 5.30	18.96 ± 2.54	<0.001
11:00 nap (min)	9.46 ± 5.73	12.57 ± 6.07	14.27 ± 5.70	18.72 ± 2.98	<0.001
13:00 nap (min)	9.11 ± 4.98	11.13 ± 6.06	14.92 ± 5.96	17.99 ± 3.74	<0.001
15:00 nap (min)	10.27 ± 5.98	14.61 ± 6.23	16.57 ± 4.90	19.02 ± 2.67	<0.001

Percent for categorical and other variables are presented with mean ± SD.

In Table 3, we present the association of insomnia and physiological hyperarousal with underweight and overweight after progressively adjusting for potential confounders. First, insomnia showed a significant association with underweight (OR = 1.78, 95% CI 1.01–3.86). Furthermore, the odds of being underweight were significantly increased among insomnia patients with increased physiological hyperarousal: insomnia with MSLT14–17 min and MSLT > 17 min increased the odds of underweight by approximately 89% (OR = 1.89, 95% CI 1.00–4.85) and 273% (OR = 3.73, 95% CI 1.51–9.22) compared with normal sleepers, respectively. In contrast, insomnia patients with MSLT 11–14 min and 8–11 min did not have significantly increased odds of being underweight. Meanwhile, insomnia was associated with decreased odds of overweight (OR = 0.44, 95% CI 0.27–0.72) instead of increased odds.

**Table 3:** Adjusted odds ratios (ORs) and 95% confidence intervals (CIs) for the association of underweight and overweight with insomnia and different levels of physiological hyperarousal.

		OR (95% CI) of underweight		OR (95% CI) of overweight	
Predictors	*n*	Unadjusted	Fully Adjusted^a^	Unadjusted	Fully Adjusted^a^
Normal sleep	185	Reference	Reference	Reference	Reference
Insomnia	440	2.31 (1.14–4.66)	1.78 (1.01–3.86)	0.43 (0.29–0.64)	0.44 (0.27–0.72)
Insomnia with different levels of physiological hyperarousal					
8 min < MSLT < 11 min	99	0.80 (0.26–2.43)	0.54 (0.16–1.80)	0.50 (0.28–0.90)	0.61 (0.31–1.22)
11 min ≤ MSLT < 14 min	118	1.95 (0.83–4.60)	1.73 (0.67–4.48)	0.43 (0.24–0.77)	0.41 (0.21–1.03)
14 min ≤ MSLT ≤ 17 min	115	2.02 (1.09–4.77)	1.89 (1.00–4.85)	0.41 (0.23–0.75)	0.39 (0.18–0.88)
MSLT > 17 min	108	4.76 (2.18–10.44)	3.73 (1.51–9.22)	0.38 (0.19–0.73)	0.36 (0.17–0.74)

^a^Adjusted for age, sex, tobacco, alcohol drinking, coffee use, diabetes mellitus, AHI, total sleep time, sleep efficiency and arousal index.

We also applied linear-regression models to examine the association between BMI, NC, WC, and MSLT as continuous variables (Table 4). MSLT was significantly associated with BMI in insomnia patients, but not in normal sleepers. In stratified analyses in Table 5, the association of BMI with MSLT was seen among men and women, younger ages, and older ages. We further conducted linear-regression models to examine the association between BMI, NC, WC, and ESS as continuous variables. ESS was only associated with BMI in normal sleepers (*β* = 0.16, *P* = 0.021) but not in insomniacs (*β* = 0.08, *P* = 0.091).

**Table 4:** Association between BMI, neck circumference, waist circumference and MSLT.

	*β*	*P*
Normal sleep		
BMI	−0.13	0.120
Neck circumference	−0.08	0.403
Waist circumference	−0.01	0.910
Insomnia		
BMI	−0.15	0.006
Neck circumference	−0.02	0.786
Waist circumference	−0.03	0.418

Adjusted for age, sex, tobacco, alcohol drinking, coffee use, diabetes mellitus, AHI, total sleep time, sleep efficiency and arousal index. Further adjusted for BMI in WC analysis.

**Table 5:** Association between BMI, neck circumference, waist circumference and MSLT in different subgroups with insomnia.

	Sex		Age	
	Men (*n* = 156)	Women (*n* = 284)	<40 y (*n* = 230)	≥40 y (*n* = 210)
BMI	−0.17^*^	−0.12^*^	−0.11^*^	−0.17^*^
Neck circumference	−0.01	−0.02	−0.01	−0.02
Waist circumference	−0.01	−0.04	−0.06	−0.02

Adjusted for age, sex tobacco, alcohol drinking, coffee use, diabetes mellitus, AHI, total sleep time, sleep efficiency and arousal index. Further adjusted for BMI in WC analysis. ^*^P < 0.05

**Fig. 1:**
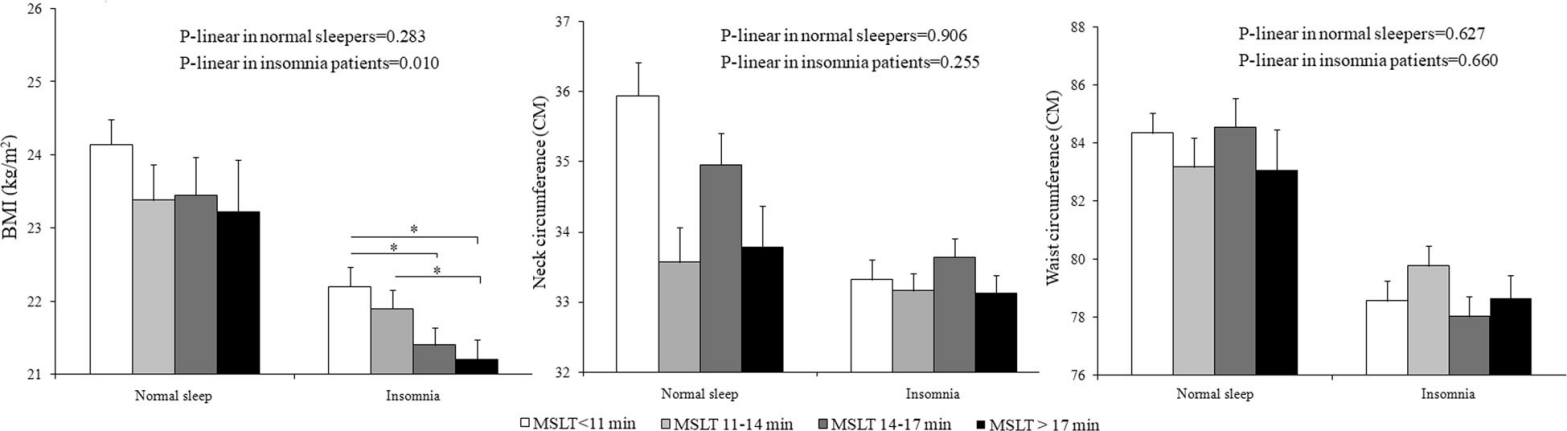
BMI, neck circumference, and waist circumference across different levels of physiological hyperarousal in normal sleepers and insomniacs. Error bars indicate standard error. MSLT indicates multiple-sleep-latency test. *P < 0.05. Analysis was adjusted for age, sex, tobacco, alcohol drinking, and coffee use, diabetes mellitus, AHI, total sleep time, sleep efficiency, and arousal index. Further adjusted for BMI for waist circumference.

Furthermore, Fig. 1 depicts the observed association between different levels of physiological hyperarousal and BMI, NC, and WC in normal sleepers and insomniacs, respectively, adjusted for confounders. Significant linear trends for BMI were obtained among insomniacs. Insomnia with MSLT > 17 min had the lowest BMI compared with those with MSLT < 14 min. However, no significant differences in BMI, NC, or WC were obtained across different levels of physiological hyperarousal in normal sleepers.

## Discussion

In this study, we investigated the association between physiological hyperarousal and BMI in insomnia patients. We report that insomnia with physiological hyperarousal, as measured by the MSLT, is associated with significantly increased odds of lower weight and even underweight, rather than overweight or obesity, in a dose–response manner. This is a novel finding not previously reported, and is in accordance with previous physiologic data that this subtype of insomnia is associated with activation of the stress system and increased basal metabolic rate, and has important medical implications. This increased risk is independent of comorbid conditions frequently associated with insomnia or BMI, such as age, sex, sleep duration, apnea–hypopnea index, diabetes mellitus, alcohol, tobacco, and caffeine use, as well as depression, anxiety, or other comorbid sleep disorders.

Given that both short sleep duration and poor sleep are considered risk factors for higher BMI [16, 17], insomnia can be expected to be associated with overweight or obesity. However, the literature on the association of insomnia with weight gain is inconsistent. The latest meta-analysis, including 67 studies, only found a fairly weak relationship between obesity and insomnia symptoms and insomnia diagnosis itself could not increase the odds of obesity [1]. Huang and colleagues [27] showed no difference between 141 patients with primary insomnia compared with normal sleepers when systematically investigating BMI in patients with insomnia. Furthermore, Cronlein et al. [20] found lower BMI in patients with insomnia, which is consistent with our study. We found that insomnia patients had an increased odds of lower weight and underweight compared with normal sleepers. Together, these studies challenged the assumption that insomnia may lead to weight gain. There could be confounding variables that can affect the association of insomnia and BMI. As the most important specific feature of insomnia, physiological hyperarousal may be the most likely mediating factor.

As discussed earlier, physiological hyperarousal could modulate associations of insomnia with several medical disorders and mortality [28]. The effect of physiological hyperarousal on BMI in insomnia has not been fully explored. Vgontzas et al. [29] showed that physiological hyperarousal measured by objective short sleep was not associated with the incidence of obesity after seven years in a longitudinal study with 812 nonobese adults, however, they found a significant association between subjective short sleep and obesity when poor sleep and emotional stress were observed. Thus, they concluded that self-reported short-sleep duration could be a surrogate marker for poor sleep and emotional stress that are related to weight gain. Our study corroborates this previous finding that physiological hyperarousal was not associated with obesity, and further shows that, compared with normal sleepers, insomnia with physiological hyperarousal was associated with underweight status in a dose–response manner.

Unlike the MSLT, ESS scores were unexpectedly not found to be associated with underweight. The correlation between ESS and MSLT is low [30–32], and the MSLT has been shown to be associated with several biological indicators, while ESS scores have not. For example, Bonnet and Arand [33] found that resting heart rate and sympathetic nervous-system activity are associated with MSLT, but not with ESS in healthy subjects. In addition, Li et al. [12] found that MSLT, but not ESS, was associated with hypertension in insomnia patients. Thus, ESS and MSLT may reflect different dimensions of nervous-system function with the MSLT being a better measurement of physiological hyperarousal.

The rate of underweight we observed in total insomnia patients was 14.1%, which appeared to be higher even compared with the average rate of underweight in Chinese population (8.1% in men and 7.8–12.6% in women) [25, 34]. Thus, underweight status seems more prevalent in the insomnia population than in the general Chinese population. Being underweight is associated with increased risk for medical disorders and mortality relative to the normal-weight category, especially when individuals suffer from severe physical illness [35–37]. For example, Dobner et al. [38] and Park et al. [39] found that underweight individuals were more vulnerable to infection and cardiovascular diseases. Funada et al. [40] showed a substantially increased percentage of hemorrhagic stroke mortality associated with underweight. A recent meta-analysis found that patients with both high and low prediagnosis BMI had higher risk for heart-failure mortality, with a greater risk from being underweight, rather than being obese [36]. Thus, more attention should be paid to the problem of being underweight associated with insomnia.

Being underweight is normally seen among patients with wasting conditions such as cancer and diabetes [41, 42]. Objective sleep disturbances evaluated by PSG appear to be a general phenomenon in these patients and those with lower BMI appear to have more obvious sleep disturbances compared with those with higher BMI [41, 43]. Among pathologic conditions associated with wasting conditions, hypermetabolism, defined as having an increased resting metabolic rate, is one of the most important [42]. Interestingly, several studies have shown increased metabolic rate in chronic insomniacs with hyperarousal when compared with normal sleepers [22]. For example, Bonnet et al. [22] found an elevated metabolic rate, as measured by overall oxygen use (VO_2_), both at night and during the day in patients with primary insomnia. Metabolic rate might be a general index of increased physiological activity. We therefore suggest that lower BMI in insomnia patients with hyperarousal may reflect a wasting condition associated with, or arising from, their hyperarousal.

While the possible mechanisms linking sleep and overweight and obesity have been well described, less is known of the pathways that may link sleep and underweight status. One possibility is that the hyperarousal state is associated with a chronic stress response [44] and a high basal metabolic rate [5], which may in turn prevent weight gain. In additionally, it has been suggested that a low calorie intake may be linked with low levels of sleep-inducing gut peptides such as cholecystokinin, as well as to an increase in wake agents such as orexin [45].

Insomnia is associated with higher cortisol level, which might also have contributed to the physiological hyperarousal [46]. However, cortisol is associated with obesity rather than underweight [47]. These findings are seemingly paradoxical; however, cortisol is also found to be associated with a higher metabolic rate [48] and hyperarousal in insomnia itself is also associated with higher metabolic rate [5]. Therefore, we speculate that this apparent paradox may be due to the fact that the impact of cortisol on obesity is not as strong as the effect of high metabolic rate on weight loss if the intake of calories stays constant in insomnia patients.

The strengths of this study include the large sample size, diagnosis of insomnia based on a standard diagnostic manual (DSM-IV-TR), and careful ascertainment of the subjects by excluding those on psychotropic medications or with frequent sleep and mental disorders. Nonetheless, some limitations should be acknowledged: (1) the results in this study were based on one PSG and MSLT recording, thus, night-to-night variation and first night effects cannot be ruled out. However, previous studies on stability of PSG and MSLT found that sleep parameters based on PSG and MSLT (e.g., total sleep time, sleep latency, and MSLT) based on three consecutive nights or two single recordings separated by eight months in both patients with insomnia and controls were stable and reflected a person’s habitual sleep [49, 50]; (2) because underweight is associated with sleep disturbances [51], our cross-sectional study cannot provide causality in terms of the direction of the association; (3) we do not have data on energy intake and physical activity that are associated with changes in BMI [42]. It is therefore possible that our findings may differ based on energy intake or physical activity status, especially when insomniacs with underweight in our study had more women and were younger subjects who are more likely to strive to be thin; (4) although we mentioned that physiological hyperarousal is associated with wasting conditions or hypermetabolism, there is no other data or biomarker evidence to support this hypothesis in our study. Thus, mechanistic studies that test the hypothesis are needed in the future; and (5) the BMI does not reflect necessarily fat mass (FM) and, FM percentage, rather than BMI, predicts some biological indices such as inflammatory and immune responses. Thus, greater accuracy should be obtained by classifying obesity condition and correlated clinical implications on the basis of body-fat composition and distribution, rather than simply on the BMI [52].

In conclusion, insomnia with physiological hyperarousal, as measured by the MSLT, is associated with significantly increased odds of lower weight and underweight in a dose–response manner. These factors suggest that insomnia with hyperarousal should be considered as a possible contributor to wasting conditions.

## Supplementary information

Supplement tables and methods
