# Supplementary material for: Insomnia with physiological hyperarousal is associated with lower weight: a novel finding and its clinical implications
Source: Transl Psychiatry. 2021 Nov 28;11:604. doi: 10.1038/s41398-021-01672-5 (PMC8628004; doi:10.1038/s41398-021-01672-5)
Supplement: Supplementary file 1 — Supplement tables and methods [file 41398_2021_1672_MOESM1_ESM.doc]

**Online Data Supplements**

**Insomnia with physiological hyperarousal is associated with lower weight: a novel finding and its clinical implications**

Rong Ren^1#^, Ye Zhang^1#^, Linghui Yang^1^, Larry D. Sanford^2^, Xiangdong Tang^1^*

^1^Sleep Medicine Center, Department of Respiratory and Critical Care Medicine, Translational Neuroscience Center, State Key Laboratory, West China Hospital, Sichuan University, Chengdu, China

^2^Sleep Research Laboratory, Center for Integrative Neuroscience and Inflammatory Diseases, Department of Pathology and Anatomy, Eastern Virginia Medical School, Norfolk, VA, USA

^#^Rong Ren and Ye Zhang contributed equally to this work

Running Title: Insomnia, physiological hyperarousal and lower weight

*Corresponding Author:

Xiangdong Tang, MD PhD

Sleep Medicine Center, West China Hospital, Sichuan University

28 Dian Xin Nan Jie, Chengdu City, Sichuan Province, China, 610041

Phone: 011-86-28 8542 2733

Email: [2372564613@qq.com](mailto:2372564613@qq.com)

**Methods**

Statistical analysis

Subject characteristics were summarized using means and standard deviations for continuous variables. Differences in sample characteristics between groups were tested by ANOVA and Mann–Whitney U tests for normally and non-normally distributed continuous variables, respectively.

**Results**

Body mass index, neck circumference and waist circumference of normal sleepers with different level of MSLT are presented in **Table S1**.

**Table S1. Body mass index, neck circumference and waist circumference of normal sleepers with different levels of MSLT.**

| Characteristics | MSLT< 11min  (n=84) | 11 min≤ MSLT< 14min  (n=44) | 14 min≤ MSLT≤ 17 min  (n=37) | MSLT> 17 min  (n=20) | P |
| --- | --- | --- | --- | --- | --- |
| Body mass index (kg/m^2^) | 24.53 ± 3.50 | 22.87 ± 3.79 | 23.41 ± 2.87 | 22.80 ± 2.88 | 0.012 |
| Neck circumference (cm) | 35.94 ± 4.50 | 33.57 ± 3.16 | 34.55 ± 3.83 | 33.78 ± 3.55 | 0.005 |
| Waist circumference (cm) | 86.59 ± 11.10 | 80.76 ± 7.91 | 83.81 ± 9.24 | 80.60 ± 9.88 | 0.004 |
